# Traumatic arteriovenous fistula mimicking cutaneous leishmaniasis: A case report

**DOI:** 10.1016/j.ijscr.2023.108334

**Published:** 2023-05-18

**Authors:** Hamed Ghoddusi Johari, Aida Bazrgar, Arshin Ghaedi

**Affiliations:** a Trauma Research Center, Vascular Surgery Department, Shiraz University of Medical Sciences, Shiraz, Iran; b Thoracic and Vascular Surgery Research Center, Shiraz University of Medical Sciences, Shiraz, Iran; c Student Research Committee, School of Medicine, Shiraz University of Medical Sciences, Shiraz, Iran; d Trauma Research Center, Shahid Rajaee (Emtiaz) Trauma Hospital, Shiraz University of Medical Sciences, Shiraz, Iran

**Keywords:** Traumatic arteriovenous fistula, Venous ulcer, Leishmaniasis, Case report

## Abstract

**Introduction:**

Traumatic arteriovenous fistula (TAVF) may be challenging to diagnose and can be misjudged as skin lesions or ulcers, including cutaneous leishmaniasis. Here, we present a patient with TAVF misdiagnosed and treated as cutaneous leishmaniasis.

**Case presentation:**

A 36-year-old male presented with a non-healing venous ulcer in his left leg, which was misdiagnosed and treated as cutaneous leishmaniasis. He was referred to our clinic, where color Doppler sonography showed arterial flow in the left great saphenous vein, and Computed tomographic (CT) angiography revealed left superficial femoral artery fistula to the femoral vein. The patient had a history of shotgun injury six years ago. Surgical closure of the fistula was done. The ulcer healed completely one month after the surgery.

**Discussion and conclusion:**

TAVF may present as skin lesions or ulcers. Our report emphasizes the importance of thorough physical examination and history taking and the use of color Doppler sonography in order to avoid unnecessary diagnostic and therapeutic modalities.

## Highlights

• Traumatic arteriovenous fistula (TAVF) may be misdiagnosed in some cases.
• Misjudgment of TAVF as cutaneous leishmaniasis has not been established before.
• Clinical examination and color Doppler sonography help avoid unnecessary diagnostic and therapeutic modalities.

## 1 Introduction

An abnormal connection between an artery and a vein is described as an arteriovenous fistula (AVF). It may either be acquired or congenital [1]. Most acquired AVFs are iatrogenic; however, they may also be traumatic. Traumatic AVFs (TAVFs) account for up to 3.9 % of all vascular injuries [2] and have been documented in several case reports and series [3,4]. TAVFs may be divided into central and peripheral categories. As peripheral TAVFs are much more prevalent, the lower extremities are the most common location for peripheral TAVFs [5]. TAVF remains a problematic pathology owing to the lack of formal recommendations in the face of diverse presentations, various regions, and different therapeutic approaches [3].

The arterial duplex scan is very sensitive in identifying AVFs, but angiography is still considered the gold standard [3]. However, diagnosis is challenging due to the wide range of clinical manifestations and is occasionally delayed [6]. These manifestations are varicosity, pain, a palpable thrill or audible bruit locally, extremity edema, or diminished or absent pulses distally [6]. Here we present the case of a patient who suffered from a TAVF due to the shotgun injury and was misdiagnosed and treated as cutaneous leishmaniasis several years after the event. This manuscript was prepared according to the SCARE 2020 guidelines [7].

## 2 Presentation of case

**Fig. 1:**
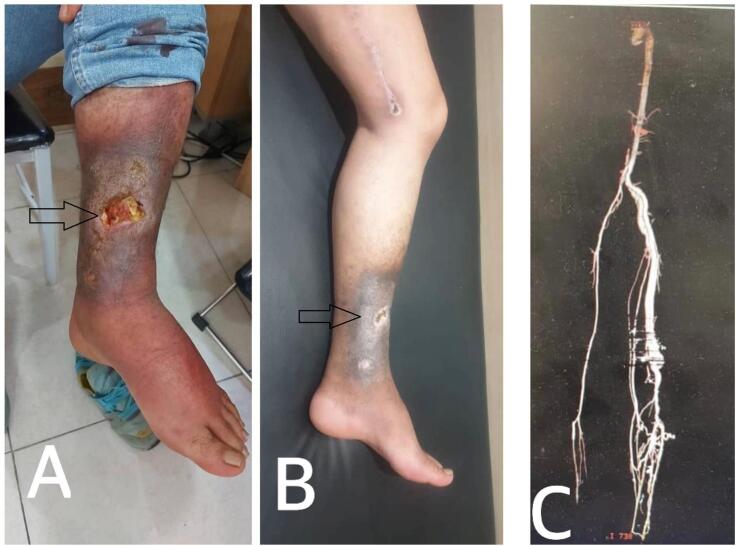
A: Left leg before surgery; B: Left leg after surgery; C: CT angiography of the left lower extremity.

**Fig. 2:**
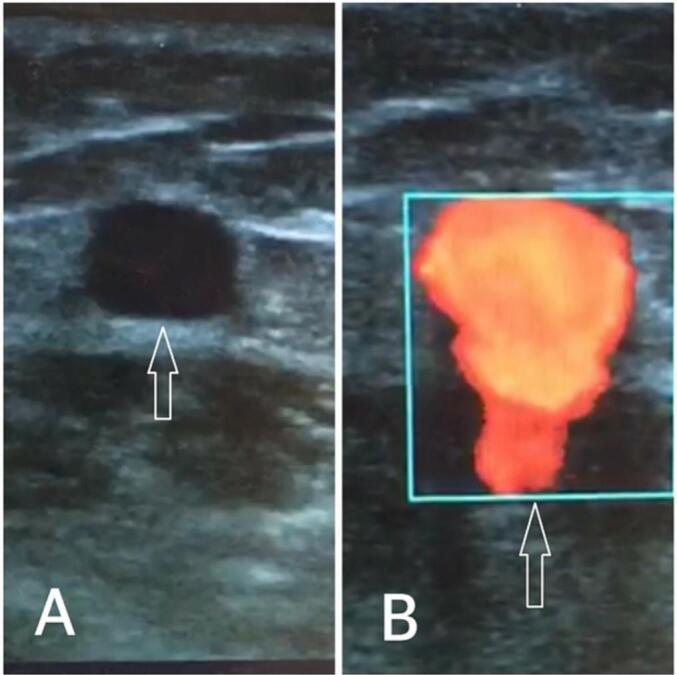
A: Great saphenous vein in color Doppler sonography; B: Arterial flow in great saphenous vein.

**Fig. 3:**
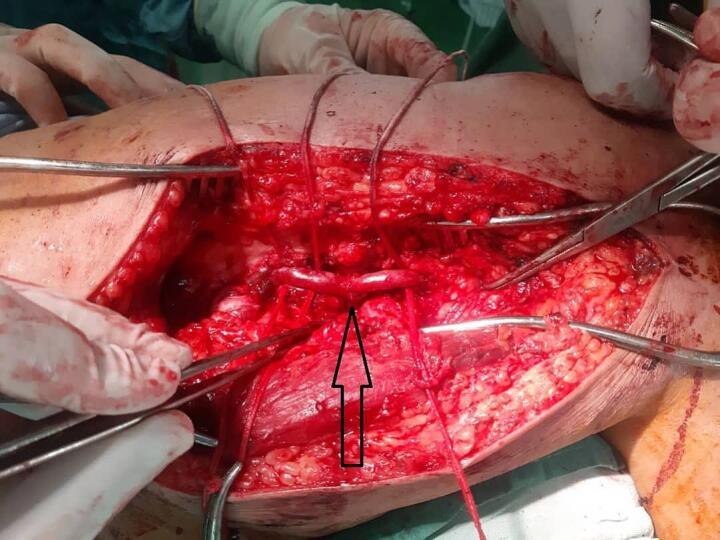
Intra-operative picture of the arteriovenous fistula.

A 36-year-old man was referred to our vascular surgery service with a chief complaint of typical non-healing venous ulcer in the medial aspect of his left leg (Fig. 1). He was symptomatic and reported pain and restrictions in movement. This wound had been treated as cutaneous leishmaniasis without any response. Because the patient suffered from cellulitis and inflammation in his leg wound, he was hospitalized and given antibiotics. Based on the patient's characteristics, color Doppler sonography of the left lower extremity venous system was done, revealing arterial flow in the left great saphenous vein (Fig. 2). The patient was asked about it, and he revealed that several years ago, he experienced a shotgun injury in his left thigh, so to rule out a traumatic arteriovenous fistula computed tomographic (CT) angiography was done and revealed the left superficial femoral artery fistula to the femoral vein (Fig. 1). Surgical closure of the fistula was done successfully including interposition saphenous vein grafting of the superficial femoral artery and repairing of superficial femoral vein (Fig. 3).

The patient had an uneventful post-op course and the ulcer healed completely after one month. Before admission, the patient required a wheelchair and could not walk properly; however, now he is mobile and symptom-free.

## 3 Discussion

Arteriovenous fistula complicates vascular trauma in 2–3 % of cases [8]. TAVF symptoms are divided into three categories: localized early signs, including pain and swelling [9]; local issues, including venous and arterial insufficiencies [9,10]; systemic problems, including pulmonary hypertension and high-output heart failure [11,12]. It is important to note that clinical manifestations sometimes arise years later, and patients often experience a chronic injury [13]. The duration and size of the fistula are two parameters that affect its consequences and its ability to be tolerated [14]. Some fistulas can go unnoticed and do not appear for several years [15]. For example, Veldhoen and colleagues described a 58-year-old man who experienced worsening pulmonary hypertension 6.5 years after being shot, which was later found to be caused by an arteriovenous fistula related to the gunshot [12]. Another study detailed a 64-year-old woman with leg swelling and a popliteal artery bruit, which was ultimately diagnosed as an AVF resulting from a stab wound 25 years prior [16]. A recent case report also highlighted a 30-year-old patient with a chronic injury to the right medial malleus, who was found to have TAVF on ultrasonography and CT angiography due to a previous gunshot injury [17]. Similarly, in our study, the patient developed a non-healing leg wound due to TAVF several years after being shot. Patients with TAVFs may get unnecessary treatment and surgeries after being misdiagnosed as having various skin lesions or cutaneous ulcers. For instance, Suknaic et al. presented a 29-year-old man who had a chronic ulcer on the outer part of his lower left leg. Angiography revealed a large AVF between femoral vein and superficial femoral artery caused by a gunshot wound to the left thigh during a war conflict 18 years prior [18]. Likewise, our patient had a persistent wound on his leg that failed to heal due to a TAVF resulting from a gunshot injury.

It is appropriate to evaluate TAVF cases carefully and completely. Clinical examination and operator-dependent ultrasonography help with the diagnosis [17]. This examination allows for direct visualization of the arteriovenous fistula but may also reveal indirect symptoms, such as a reduction in downstream arterial flow [17]. Additionally, the vein's spectrum is arterialized [19]. However, the arteriovenous fistula's specific topography and shape cannot be determined [20]. This expedites both the diagnosis and the course of therapy [17]. The location, topography, and shape are described by CT angiography. Usually, this examination is enough to determine the diagnosis and the best course of treatment [13]. However, angiography is sometimes mandatory due to bullet artifacts and enables an endovascular treatment.

Endovascular or surgical procedures are the therapeutic approaches for an arteriovenous fistula. The first has the advantages of a quicker procedure, lower risk of bleeding, less postoperative discomfort, lower [6] duration of hospitalization, and fewer complications. The technique entails inserting “stents,” or “coils”. However, there are some restrictions, such as size mismatch or risk of stent fracture at joint levels [21]. Additionally, we may mention the danger of stent misplacement, limb ischemia, pulmonary embolism, and endoleak [17,22]. Only in cases where endovascular therapy is not possible surgery is advised, such as in our case where the distal landing zone was at knee level, so the surgical approach was selected. Surgical treatment consists of ligation and resection of AVF and then anastomosis of veno-venous and arterio-arterial [14,23]. When circumstances do not permit, a venous or prosthetic bypass is recommended to prevent venous insufficiency [24]. It should be noted that long-term venous bypasses have higher permeability than prostheses.

## 4 Conclusion

There have been reports of misdiagnoses of TAVF before, but this study was the first which reported TAVF can mimic cutaneous leishmaniasis. The patient suffered from a non-healing venous ulcer in his leg several years after the shotgun injury in his left thigh and was misdiagnosed as cutaneous leishmaniasis. This study and prior case reports on TAVF misdiagnoses support the importance of history taking, physical examination, and the use of color Doppler sonography and vascular imaging in order to avoid further misdiagnoses and unnecessary diagnostic and therapeutic modalities. Our study also emphasizes that patients who sustain gunshot injuries must be followed up with the possibility of TAVFs.

## Consent

Written informed consent was obtained from the patient for publication of this case report and accompanying images. A copy of the written consent is available for review by the Editor-in-Chief of this journal on request.

## Ethical approval

Ethical approval is exempt/waived at our institution for this study.

## Funding

This research did not receive any specific grant from funding agencies in the public, commercial or not-for-profit sectors.

## Author contribution

**H.Gh.J:** Conceptualization, Methodology, Writing – review & editing, Visualization. **A.B:** Investigation, Writing original draft. **A.Gh:** Writing – review & editing, Supervision, Project administration.

## Guarantor

A.Gh.

## Research registration number

Not applicable.

## Availability of data and materials

All data regarding this study has been reported in the manuscript. Please contact the corresponding author if you are interested in any further information.

## Consent for publication

Written informed consent was obtained from the patient for publication of this case report and any accompanying images. A copy of the written consent is available for review by the Editor-in-Chief of this journal.

## Conflict of interest statement

None to declare.
